# Quality and Consumer Acceptance of Meat from Rabbits Fed Diets in Which Soybean Oil is Replaced with Black Soldier Fly and Yellow Mealworm Fats

**DOI:** 10.3390/ani9090629

**Published:** 2019-08-29

**Authors:** Laura Gasco, Sihem Dabbou, Francesco Gai, Alberto Brugiapaglia, Achille Schiavone, Marco Birolo, Gerolamo Xiccato, Angela Trocino

**Affiliations:** 1 Department of Agricultural, Forest and Food Sciences, University of Turin, Largo Paolo Braccini 2, 10095 Grugliasco, Italy; 2 Department of Veterinary Science, University of Turin, Largo Paolo Braccini 2, 10095 Grugliasco, Italy; 3 Institute of Science of Food Productions, National Research Council, Largo Paolo Braccini 2, 10095 Grugliasco, Italy; 4 Department of Agronomy Food Natural Resources Animal and Environment (DAFNAE), University of Padova, Viale dell’Università 16, I-35020 Legnaro, Padova, Italy; 5 Department of Comparative Biomedicine and Food Science (BCA), University of Padova, Viale dell’Università 16, I-35020 Legnaro, Padova, Italy

**Keywords:** proximate chemical composition, lipid peroxidation, sensory analysis, insect lipids, dietary oils

## Abstract

**Simple Summary:**

Insect lipids are an interesting, potential feed ingredient for animal farming and maybe a suitable alternative to less eco-friendly fat sources (i.e., soybean, palm kernel, coconut, and fish oil). The possible utilization of insect fats in rabbit diets has been poorly investigated so far, and only a few papers that show encouraging results, in terms of growth performance, diet digestibility, and the intestinal morphology of rabbits, are available. The present study evaluated the effect of a partial (50%) or total (100%) substitution of soybean oil by two insect fats (Black soldier fly, H and Yellow mealworm, T) on the characteristics, proximate composition, lipid peroxidation, and fatty acid profile of the meat of rabbits, as well as on consumer acceptance. The results are encouraging as the meat of the rabbits fed the diets containing insect fats was less susceptible to oxidation, while the meat of the rabbits given diets with H fat had higher concentrations of saturated fatty acid rate and a lower polyunsaturated fatty acid rate than those fed the diets with soybean and T fat. Overall, these results highlighted the possibility of replacing soybean oil with H and T fats in rabbit diets without affecting consumer acceptance.

**Abstract:**

This trial investigated the effect of the dietary inclusion of *Hermetia illucens* (H) and *Tenebrio molitor* (T) fats as alternative lipid sources for growing rabbits, and assessed the carcass characteristics; proximate composition; lipid peroxidation, and fatty acid profile of the meat, as well as consumer acceptance. At weaning, 200 crossbred rabbits (1051 ± 138 g initial body weight) were allotted to five isolipidic (4% dry matter (DM)) dietary treatments: a control diet (C) containing 1.5% of soybean oil, and four experimental diets in which soybean oil was partially (50%) or totally (100%) substituted by H (H50 and H100) or T (T50 and T100) fats. The carcass characteristics, the meat quality traits, and the consumer acceptance of the cooked meat were not affected. The fat content of *Longissimus thoracis* et *lumborum* muscle of the rabbits was 1.1% on average. In the case of rabbit fed the H diets (average of diets H50 and H100), the same muscles revealed a higher saturated fatty acid proportion (47.1% vs. 39.7% and 40.8%, respectively) and a lower polyunsaturated fatty acid proportion than the rabbits fed the C and T diets (average of diets T50 and T100) (26.5% vs. 31.7% and 29.7%) (*p* < 0.001). The meat of the rabbits fed the diets containing insect fat (average for H and T diets) was less susceptible to oxidation (0.24 vs. 0.39 mg malondialdehyde/kg meat in the C group; *p* < 0.01).

## 1. Introduction

In some Mediterranean countries, such as Italy, Spain, and France, the rabbit is a traditional meat product, although it is currently suffering from consumption regression [1]. Rabbit meat is promoted as a healthy and nutritious alternative to beef and pork [2]. Its fat composition is characterized by low levels of saturated fatty acids (SFA) and a good proportion of polyunsaturated fatty acids (PUFA; 35–40% of the total fatty acids; FA), which is significantly higher than other meats [2]. However, several factors may influence the meat quality of rabbits, especially the quantity and quality of the lipids in the diet. The fat source can affect fat absorption, the FA composition, and, thus, the main characteristics of rabbit meat due to differences in the length of the FA chain, the degree of saturation, and the degree of esterification [3].

Insects, which have recently been used as alternative sources of protein [4,5] and lipids [6,7,8,9,10,11,12] in animal feeds, seem to be promising replacers for conventional feedstuffs [13]. Insect lipids may be suitable alternatives to resource-intensive and more expensive soybean (S) oil, palm kernel oil, coconut oil, and fish oil [10]. The FA compositions and amounts of lipids in insects vary with species, sex, stage of development, rearing substrates, and processing methods [14], with the lipid content and FA composition of Black soldier fly (*Hermetia illucens* L.; H) and Yellow mealworm (*Tenebrio molitor* L.; T) having been characterized. H lipids vary from 15 to 49% on a dry matter (DM) basis [15], with SFA making up 44.5% of the total FA, and being the main group [15]. Lauric (C12:0) and palmitic (C16:0) acids are the most abundant medium-chain SFAs in H fat, followed by myristic (C14:0), oleic, and linoleic acids [11,13,14]. The total lipids of T can reach 43% of DM [15]. Oleic (C18:1 c9), linoleic (C18:2 n6), and palmitic (C16:0) acids are the predominant FAs in T fat [10,15]. Because of its high linoleic acid content (~39% of the total FAs), T fat may be considered a source of n-6 PUFA [15]. H and T fats could represent valid alternatives to conventional lipid sources in chicken and fish diets due to their energy and essential FA content [9,11,12,15]. Dalle Zotte et al. [7] have published information about hind leg meat of rabbits fed diets containing H fat, but there is no information about the impact of T fat on rabbits, and no investigations have been carried out to assess consumer acceptance of the meat of rabbits fed diets containing insect fats.

Thus, this study aimed to investigate the effects of the dietary inclusion of *Hermetia illucens* fat or *Tenebrio molitor* fat on the carcass characteristics, proximate composition, lipid peroxidation, and FA profile of the meat of rabbits as well as on consumer acceptance, while a previous study [9] described the effects on growth performance, apparent digestibility, gut mucosa traits, and health.

## 2. Materials and Methods

### 2.1. Animals and Experimental Design

The study was conducted at the experimental rabbit facility of the Department of Agricultural, Forest, and Food Sciences (DISAFA) of the University of Turin (Italy) and was approved by the Ethical Committee of the University of Turin (Italy) (Ref. 386638, 4/12/2017).

At 36 days of age, 200 crossbred rabbits (Hycole, France −1051 ± 138 g live weight) were housed in individual wire-net cages (41 × 30 × 28 cm height) and randomly allotted to five dietary treatments (40 rabbits/group): a control diet (C) containing 1.5% of soybean oil, and four experimental diets in which soybean oil was partially (50%) or totally (100%) substituted by H (H50 and H100) or T (T50 and T100) fats. The rabbits were fed *ad libitum*. The test diets were the same as those used in the companion paper (Gasco et al. [9]), which presents additional data collected during this large study; information about the diet formulations and their compositions is given in Table 1 and Table 2.

### 2.2. Slaughtering Procedures and Sampling

At 78 d of age, 20 rabbits from each dietary treatment, which were representative of the experimental groups in terms of average live weight at slaughtering (SW) and variability, were weighed, electrically stunned, and slaughtered in a commercial slaughterhouse without fasting. The carcass dissection procedures were based on the World Rabbit Science Association (WRSA) recommendations [16]. Hot commercial carcasses (with the head, thoracic organs, liver, kidneys, perirenal, and scapular fat) were weighed and then chilled and kept at + 4 °C for 24 h in a ventilated room. The chilled carcasses (CC) were weighed, and the head, thymus, trachea, esophagus, heart, lungs, liver, and kidneys were removed to obtain the reference carcasses (RC). The dressing out (CC as a percentage of SW) and the proportions of the head, liver, and carcass parts to CC or RC were calculated.

The *Longissimus thoracis et lumborum* (LTL) muscle of 15 rabbits per dietary treatment was removed from both the left and right sides of the carcasses. The cranial portion of the left LTL muscle was used to measure the pH and color of the meat. The medial and caudal portions were vacuum-packed and frozen at −80 °C until the chemical composition and FA analyses were performed. The right LTL muscle was divided into two parts, and each of them was weighed, vacuum-packed, frozen, and stored at −20 °C for subsequent analysis. The fore part was used for thiobarbituric acid-reactive substance (TBARS) analysis. The hind part was used to measure thawing and cooking losses, as well as the Warner-Bratzler shear force.

The right and left LTL muscles of the remaining 5 rabbits from each dietary group were sampled and used for the sensory analysis.

### 2.3. Meat Quality Traits

The ultimate pH (at 24 h post-mortem) was measured at the cranial end of the left LTL muscle using a Crison portable pH-meter (Crison 507, Crison Instruments, S.A., Alella, Spain) equipped with a glass electrode suitable for meat penetration. The color was measured on the surface of the left LTL using a portable Chroma Meter CR-400 Konica Minolta Sensing colorimeter (Minolta Sensing Inc., Osaka, Japan). The device was set with a CIE (Commission Internationale de l’Éclairage) 2° standard angle observer and D65 illuminant. CIELAB coordinates [17], and the lightness (L *), redness (a *), and yellowness (b *) were recorded. The Hue angle (H *) and Chroma (C *) were calculated as H * = tan ^−1^ (b */a *) and C * = (a *^,2^ + b *^,2^)^0.5^, respectively. Three random readings were taken at different locations on the meat surface and averaged. Thawing losses were determined on the hind part of the right LTL muscle as the difference in weight of a meat sample before and after thawing and were expressed as a percentage of the initial sample weight. Cooking losses were determined as the difference in weight of a thawed meat sample before and after cooking and were expressed as a percentage of the initial sample weight. The samples were cooked for 1 h in a water bath set at 80 °C [18].

The cooked samples were also used for the Warner Bratzler shear force test. Three cores (~3–4 cm long, 1 cm^2^ cross-sectional) obtained from each sample were cut perpendicularly to the longitudinal orientation of the muscle fibers with a V-shaped cutting Warner-Bratzler blade, which was fitted to an Instron Universal Machine, model 5543 (Canton, MA, USA). Tenderness was measured as the maximum force (Newtons) required to shear the core at a crosshead speed of 200 mm/min [19].

### 2.4. Proximate Composition and FA Profile of the LTL Muscle

The medial and caudal portion of the left LTL muscle was homogenized and divided into two parts. The first part was used to determine the moisture and ash contents, according to AOAC methods 950.46 and 920.153, respectively [20]. The second part was freeze-dried (Edwards MF 1000, Milano, Italy) and then analyzed to establish the protein and fat contents. The protein content was determined by means of the Kjeldahl method (method 928.08; [20]), using a Büchi Distillation Unit K-355 (Flawil, Switzerland). The fat content was determined by means of Soxhlet extraction (method 991.36; [20]), using a Büchi Extraction System B-811 (Flawil, Switzerland).

Moreover, an aliquot of fresh minced meat from the medial and caudal portions of the left LTL was analyzed to establish the fatty acid composition. The fat was extracted by means of accelerated solvent extraction (ASE^®^, Dionex, Sunnyvale, CA, USA, Application Note 334) using two extraction cycles, with petroleum ether as a solvent, at a temperature of 125 °C and at a pressure of 10.3 Mpa, with a 6-min heating phase and a 2-min extraction phase. The extracted lipids were initially trans-methylated as fatty acid methyl esters (FAMEs), using a solution of 1 M sodium methoxide in methanol (1 vol) and a solution of oxalic acid in diethyl ether [21]. An internal standard (13:1 methyl ester) was added to the extracts before methylation. After centrifugation, the supernatant was submitted to two-dimensional Gas Chromatography (GC × GC) using an Agilent 7890A Gas Chromatograph (Agilent Technologies, Santa Clara, CA, USA), with the split at 40 mL/min and the rate set at 160:1. Supelco SP 2560 (Sigma-Aldrich, St. Louis, MO, USA) was used as the first capillary column (75 m × 0.18 mm internal diameter, 0.14 μm film thickness), with hydrogen as the carrier at 0.25 mL/min. J & W HP 5 ms (Agilent Technologies, Santa Clara, CA, USA) was used as the second capillary column (3.8 m × 0.5 mm internal diameter, 0.25 μm film thickness), with hydrogen as the carrier at 22 mL/min for 2 min and then 0.18 mL/min to 35 mL/min. The oven temperature was set at 45 °C, held for 2 min, raised to 170 °C at the rate of 50 °C/min, held for 25 min, raised to 240 °C at the rate of 2 °C/min and held for 16 min, while the injector and the detector temperatures were set at 270 °C and 250 °C, respectively. The fatty acids were identified by comparing the retention time of a 52 standard FAME mixture (GLC reference standard: 674; Nu-Chek Prep, Inc., MN, USA). Individual FAMEs were expressed as the percentage of the total area of the eluted FAMEs.

The average percentage of each FA was used to calculate the atherogenicity (AI), thrombogenicity (TI), and peroxidability (PI) indexes, according to Dal Bosco et al. [22], as follows:

AI = (C12:0 + 4 × C14:0 + C16:0)/[(Σ MUFA + Σ n-6) + Σ n-3)];(1)

TI = (C14:0 + C16:0 + C18:0)/[(0.5 × Σ MUFA + 0.5 × Σ n-6 + 3 × Σ n-3) + (Σ n-3)/Σ n-6)];(2)

PI = (% monoenoic × 0.025) + (% dienoic × 1) + (% trienoic × 2) + (% tetraenoic × 4) + (% pentaenoic × 6) + (% hexaenoic × 8);(3)

where MUFA is the monounsaturated fatty acids.

### 2.5. Lipid Peroxidation

Lipid peroxidation was determined on the forepart of the right LTL samples (10 g) after 30 days of freeze storage, using a TBARS assay [23]. The samples were analyzed in duplicate, and the absorbance was read at 532 nm on a Helios spectrophotometer (Unicam Limited, Cambridge, UK). The TBARS values were calculated from a standard curve of 1,1,3,3-tetramethoxypropane (Sigma–Aldrich, Steinheim, Germany) and expressed as mg of malondialdehyde (MDA)/kg meat.

### 2.6. Consumer Test

A total of 120 untrained consumers were recruited from among the participants in a one-day conference held at the Department of Agricultural, Forest, and Food Sciences of the University of Turin. Information regarding demographics and consumption habits was collected via a questionnaire before the sensory assessment of the samples.

The sensory evaluation was conducted just before lunch, at noon, in the conference room. Each participant was provided with a score sheet with a 9-point hedonic scale to be completed for each sample from the 5 dietary treatments, a pen, a toothpick, a paper towel, and a glass of deionized water to cleanse their mouths after tasting each sample. Before the start of the test, the panelists were given verbal instructions about the test and were asked to read and sign an informed consent form.

The right and left loins collected from rabbits of the five dietary groups were thawed at 4 °C for 24 h before the assessment. Packages containing loins were opened, and the entire loins were placed in disposable aluminum pans on a wire rack, covered with aluminum foil, and cooked for ~40 min without salt or spices in a preheated convection electric oven at 150 °C until an endpoint temperature of 70 °C was reached. After cooking, the loins were immediately cut into 1 cm^3^ cubes, randomly placed (one per each treatment) on a round plastic plate, and divided into 5 equally-sized wedges. Each consumer assessed 5 samples, one from each of the dietary treatments. Random three-digit numbers were utilized to identify the samples.

The participants were asked to score the overall acceptability of the samples using the 9-point hedonic scale. The category definitions were as follows: 1 = dislike extremely; 2 = dislike very much; 3 = dislike moderately; 4 = dislike slightly; 5 = neither like nor dislike; 6 = like slightly; 7 = like moderately; 8 = like very much; 9 = like extremely [24].

### 2.7. Statistical Analysis

Statistical analysis was performed using the SPSS software package (version 21 of Windows, SPSS Inc., Chicago, IL, the USA). Shapiro–Wilk’s test was used to check the assumption of normality. One-way ANOVA was used to evaluate the effect of dietary treatment on the carcass characteristics, meat quality traits, and FA profile. The assumption of equal variances was evaluated using Levene’s variance homogeneity test. The differences between groups were evaluated using Duncan’s test.

The results of the consumer test were summarized using box-plots, which graphically represent the descriptive statistics of overall acceptability. A mixed ANOVA model was performed, with the overall acceptability as the dependent variable, the dietary treatment as the fixed effect, and the consumer and the interaction between consumer and dietary treatment as random effects, to determine any significant differences between treatments [25]. The overall acceptability scores (1 to 9) given by each consumer to the evaluated samples were converted in ordered data by assigning rank order numbers (the highest and smallest score get a rank of 1 and 5, respectively). Ties received equal fractional numbers. The sums of the ranked scores of each treatment were analyzed using Friedman’s test [26]. Internal preference mapping was applied to the hedonic data matrix, which consisted of the rabbit meats (objects) and consumers (subjects), to obtain a single bi-dimensional map, based on the meat type acceptability information obtained from each consumer [27]. To make the internal preference map results easier to interpret, a Hierarchical Cluster Analysis was conducted using Ward’s method and the Euclidean distance, to group the consumers according to their preferences. All the data obtained from the sensory test were statistically analyzed using XLSTAT (Addinsoft USA, New York, NY, version 2016.01.26136). The results were reported as means and as the standard error of the means (SEM). Significance was declared at *p* < 0.05.

## 3. Results

### 3.1. Carcass Characteristics, Meat Quality Traits, and Chemical Composition

Dietary insect fat inclusion did not influence carcass characteristics (Table 3). The average slaughter weight, chilled carcass weight, and dressing out percentage were 2906 g, 1658 g, and 57%, respectively, for all the groups (Table 3).

The same results were observed for the meat quality traits and chemical composition of the LTL muscle of rabbits fed the different lipid sources (Table 4). The protein and fat contents were on average 22.5% and 1.09%, respectively, for the five groups (Table 4).

### 3.2. Fatty Acid Profile of the LTL Muscle

The partial or total substitution of soybean oil (S) with insect lipids influenced the FA profile of the rabbit meat and gave significant differences in the proportions of most FAs in the LTL muscle (Table 5). The C12:0 proportion was higher (1.22% and 2.53% of the total FAs) in the muscles of the H50 and H100 groups than in those of the C and T, respectively. This difference was also noted for C14:0 and C16:0, which increased the proportion of total SFAs in the muscles of the rabbits fed the H diets compared to those fed the C and T diets. The proportion of the total branched-chain fatty acids (BCFAs) was also higher (*p* < 0.05) in the H50 and H100 groups than in the other groups, whereas the MUFA and PUFA proportions were lower (*p* < 0.001).

C18:1 was the main FA in the meat of the T groups (23.1% and 24.8% of the total FAs for T50 and T100, respectively). C18:2 n6 was the most abundant PUFA, with a higher concentration in the C and T50 diets (27.7 and 26.3% of the total FAs for C and T50, respectively) than in the other groups. As far as C18:3 n3 is concerned, a higher proportion was measured in the meat obtained from the animals fed diets containing 50% of H or T fats, whereas the low proportions of the long-chain n-3 PUFAs (C20:5 n3 and C22:6 n3) did not differ for the dietary treatments.

### 3.3. Dietary Indexes and Lipid Oxidation

The lipid dietary indexes were affected by the dietary treatment (*p* < 0.001), with the highest atherogenicity index, AI (1.17), and thrombogenicity index, TI (1.31), values and the lowest peroxidability index, PI (29.9), values being recorded in the H100 group. After one month of frozen storage, the average TBARS values of the MDA in the insect fat supplemented groups were affected by H and T supplementation, regardless of the levels of S replacement (Table 5).

### 3.4. Consumer Test

In our test, the consumers—43% males and 57% females—were generally aged between 18 and 24 (52%). They had a high education level (75% had a university degree) and were usual meat-eaters, i.e., they ate meat 2–3 times a week in 79% of the cases.

**Figure 1:**
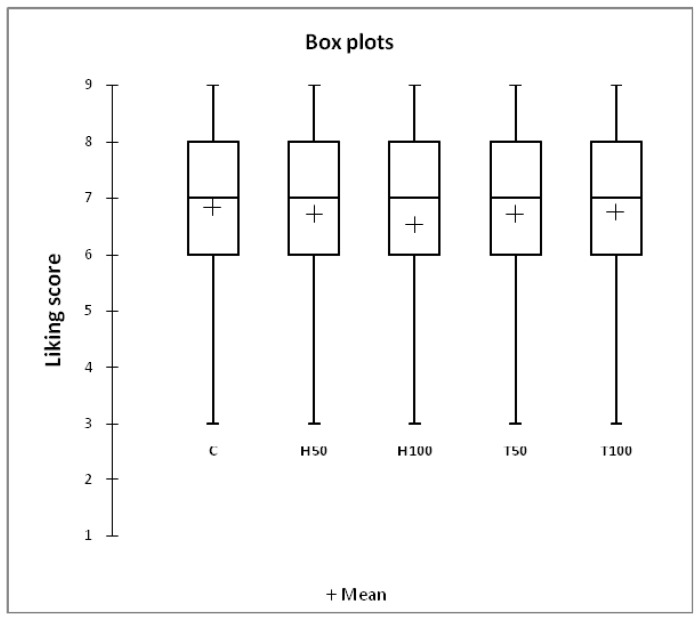
Box-plots for the overall acceptability: Evaluation on a nine-point hedonic scale. Note: C = control diet; H = *Hermetia illucens* fat; T = *Tenebrio molitor* fat. The horizontal axis represents the five dietary treatments, and the vertical axis represents the like score values. The crosses correspond to the means. The central horizontal bars are the medians. The lower and upper limits of the boxes are the first and third quartiles, respectively. The ends of the vertical lines or “whiskers” indicate the minimum and maximum like scores.

The box-plots for the overall acceptability of the meat of rabbits fed different experimental diets are presented in Figure 1. The average acceptability scores ranged from 6.5 (H100 group) to 6.8 (C and T100 groups), corresponding to the “like moderately” category according to the nine-point hedonic scale.

The average acceptability scores did not show any differences between treatments (*p* = 0.621; Table 6). When rank order numbers were used, the meat samples obtained from rabbits fed H50 showed a rank-sum of 377.5, and this was followed by H100 (rank-sum = 371), T50 (rank-sum = 357.5), T100 (rank-sum = 346.5), and the C group (rank-sum = 347.5) without any differences between treatments (*p* = 0.557) (Table 6).

## 4. Discussion

The experimental diets were formulated to be isoenergetic and isoproteic and to contain the same ingredients to avoid any possible confounding effects because of the use of different ingredients and/or different energy levels due to lipid source substitution. The partial or total substitution of S with H and T changed the total content of the different FA classes in the experimental diets according to the lipid source and the replacement rate.

In our trial, the changes in the fatty acid composition of the experimental diets, as a result of the dietary inclusion of H and T fats, did not affect the performance, as described in detail by Gasco et al. [9]. Thus, no effect was recorded for the carcass traits, according to Xiccato [28]. The results of the current study are also in agreement with those of Martins et al. [29], who did not find any significant differences in the slaughter weight or fat depots of growing rabbits fed diets with two lipid sources (H fat and linseed oil) at two inclusion levels (3 and 6%).

In the current study, the meat quality traits and chemical composition were not affected by the partial or total replacement of S with either H or T. However, Dalle Zotte et al. [7] have recently reported a higher a * index in the *Longissimus lumborum* muscle of rabbits fed H fat than in those fed linseed oil at two inclusion levels (3 and 6%). As far as the other lipid sources are concerned, neither Rodríguez et al. [30], using fish oil, nor Peiretti et al. [31], using vegetable oils, found any relevant differences in the main carcass traits or meat quality of growing rabbits.

The meat FA profile of rabbits can be modified by dietary fat [32]. For example, dietary fish oil supplementation has been found to enhance the content of the beneficial long-chain n-3 FAs and to reduce the n-6/n-3 ratio of rabbit meat and fat [30].

As far as the use of insect fats is concerned, significant differences have been recorded in the present trial between the dietary treatments. The use of H fat increased the proportion of total SFAs and reduced those of MUFA and PUFA in the muscles of the rabbits compared to the use of S oil or T fat. Overall, our results are in agreement with the findings of Dalle Zotte et al. [7] and Schiavone et al. [11] for H-fed rabbits and broiler chickens, in terms of increased SFA and decreased PUFA. The increased C16:0 content for the H diets could be considered detrimental from a health point of view since this FA has been associated with elevated plasma concentrations of total cholesterol and the low-density lipoprotein fraction in humans [33].

Indeed, the nutritional quality of fats for human consumption is generally evaluated in terms of the n-3 FA, PUFA/SFA, and Σ n-6/Σ n-3 FA ratios. In all the treatments considered in the current study, both the PUFA/SFA ratio (optimal values ≥0.45) and the Σ n-6/Σ n-3 FA ratio, which indicates a balance between the essential FAs in the meat, always fell within the optimal values for human consumption [2].

Additionally, the AI and TI indexes consider the different effects that single FAs might have on human health and, in particular, on the probability of increasing the incidence of pathogenic conditions, such as atheroma and/or thrombus formation. The addition of H fat and T oil led to a worsening of these indexes. Dalle Zotte et al. [7] showed that all the nutritional indexes measured for rabbit hind leg meat were impaired by increasing H inclusion levels. On the other hand, Kierończyk et al. [10] observed that both the AI and TI values of broiler chicken breast were reduced by dietary T oil inclusion. The highest value of both indexes in the rabbits fed the H100 diet might be related to its C12:0 content, as previously found by Schiavone et al. [11] for the breast meat of H-fed chickens.

After one month of frozen storage, the meat from the rabbits of the H and T supplemented groups showed lower susceptibility to lipid peroxidation than that of the S group. Consistently with our results, other authors have shown that, after 6 weeks of frozen storage, the meat of rabbits fed H presented lower TBARS values than the meat of rabbits fed linseed oil-enriched diets [7].

A consumer acceptance test was performed to determine whether there was a discernible difference in meat acceptability for the different dietary treatments. To this aim, a sensory evaluation using untrained consumers, which has been reported to be an effective alternative to trained taste panels [34,35], was performed. In this case, consumer taste panels require several participants due to the higher level of variation associated with untrained consumers, but they provide relatively unbiased results that are relevant for the meat industry [36].

The medians of all the meat groups were very close to the average acceptability scores, and a median value of seven (like moderately) was obtained for all the groups. A mean like the score of seven or higher on a nine-point scale is usually indicative of highly acceptable sensory quality. None of the consumers chose the first two categories, and all the samples resulted in most of the choices falling into the “like part” (consumer ratings >5) of the hedonic scale. On average, 80% of the consumers rated the meat of the five groups equally acceptable.

**Figure 2:**
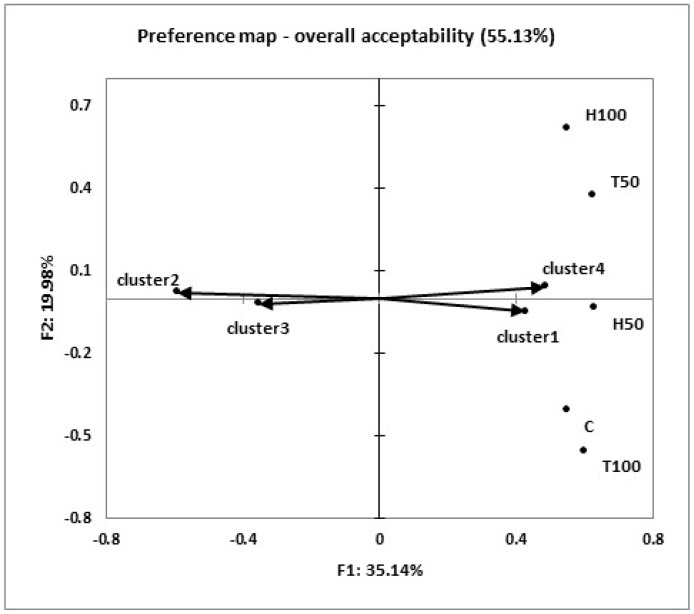
Internal preference map of the overall acceptability. Each preference vector (→) represents a cluster group of the panelist. Note: C = control diet; H = *Hermetia illucens* fat; T = *Tenebrio molitor* oil.

Moreover, the consumers were grouped into four homogeneous clusters according to their preferences, and an internal preference map was used to create an intuitive and “easy to read” visual representations (Figure 2). The results of the internal preference analysis showed that two preference dimensions explained 55.13% of the data variance (Figure 2). An examination of the preference scores within each of the four clusters revealed a quite similar preference trend. The consumers from cluster one (n = 41), corresponding to 34% of all the panelists, provided higher scores and preferred meat from the H50, T50, C, and T100 groups, with a mean acceptance score of ≥7 (like moderately). The consumers in cluster four (n = 16) showed a marked preference for H100, H50, and T50, in increasing order of preference, with a mean acceptance score of eight. The consumers in cluster two (n = 41; 34% of the panelists), which is located in the higher negative half of the first dimension and opposite H50 and T100, indicated a relative lower preference for them, with a mean acceptance score >6. Cluster 3 (n = 22; 18% of the panelists), located in the lower negative half of the first dimension, opposite T50, confirmed the consumers’ relatively low acceptability of this meat (mean acceptance score = 5).

The replacement of S oil with H fat in chicken diets did not affect the meat sensory evaluation [6]. The inclusion of H larva meal in the diets of rainbow trout [36,37] or quail [38] did not affect the sensory evaluation of either trained or untrained panelists. Small differences were only reported for some sensory traits of meat in chickens fed *Musca domestica* larva meal [39].

To the best of our knowledge, this is the first study that assessed consumers’ acceptability of meat obtained from rabbits fed diets supplemented with insect fats. Our results preliminarily support the possibility of a market for this kind of meat, but require further confirmation through the use of panels of different composition (mainly concerning age and education background).

## 5. Conclusions

This study provided new insights into the use of insect lipids in rabbit diets and into the implications of their inclusion on meat quality and consumers’ acceptance. The partial or total replacement of S sources with H or T lipids in commercial diets for growing rabbits could be introduced with no detrimental effects on the main carcass and meat rheological traits, that is, for the standard level of fat inclusion. Moreover, the first results showed that consumers (usual meat-eaters) were not able to distinguish between the meat of rabbits fed vegetable fat and those fed insect fat. However, the use of insect fats increased the SFA proportion and the n-6/n-3 PUFA ratio of rabbit meat, especially for H fat, which is considered a potential negative consequence on the nutritional value of the meat. For this reason, the use of insect fats in rabbit diets requires a careful modulation of the FA profile of the insects, which might be obtained using suitable rearing substrate for insects and fat extraction processes, to increase the level of healthy FAs, such as n-3 PUFAs.

## Figures and Tables

**Table 1:** Ingredients (% as fed) and chemical composition (% DM) of the experimental diets (modified from Gasco et al. [9]).

Items	Experimental Diets				
	C	H50	H100	T50	T100
Dehydrated alfalfa meal (17 g CP/100 g)	32	32	32	32	32
Alfalfa hay	7.5	7.5	7.5	7.5	7.5
Wheat bran	23.5	23.5	23.5	23.5	23.5
Barley meal	10	10	10	10	10
Dried sugar beet pulp	16	16	16	16	16
Soybean meal (44 g CP/100 g)	7	7	7	7	7
Soybean oil	1.5	0.75	-	0.75	-
*Hermetia illucens* fat	-	0.75	1.5	-	-
*Tenebrio molitor* fat	-	-	-	0.75	1.5
Cane molasses	1.2	1.2	1.2	1.2	1.2
Dicalcium phosphate	0.3	0.3	0.3	0.3	0.3
Sodium chloride	0.4	0.4	0.4	0.4	0.4
L–methionine (98 g methionine/100 g)	0.1	0.1	0.1	0.1	0.1
Vitamin-mineral premix ^a^	0.5	0.5	0.5	0.5	0.5
Chemical Composition					
Dry matter (%)	89.4	90	89.2	89.5	89.6
Ash (% DM)	8.58	7.67	7.77	8.18	7.75
Crude protein (% DM)	17	16.4	16.8	16.8	16.3
Ether extract (% DM)	4.22	4.07	3.92	4.13	3.87
Neutral detergent fiber (aNDF) (% DM)	40.2	42.5	41.7	39.8	40.5
Acid detergent fiber (ADF) (% DM)	21.7	23.8	23	21.4	22.8
Acid detergent lignin (ADL) (%DM)	4.81	5.09	5.09	4.87	5.02
Gross Energy (MJ/kg DM)	18.50	18.63	18.50	18.75	18.62

C, control diet; H50 and H100, diets with *Hermetia illucens* fat; T50 and T100, diets with *Tenebrio molitor* fat; DM, dry matter; CP, crude protein; ^a^ Premix provided per kg of complete diet: vitamin A 16000 UI; vitamin D3 1600; vitamin E acetate 30 mg; vitamin B1 0.8 mg; vitamin B6 1.65 mg; niacin 40 mg; folic acid 1 mg; Mn 30 mg; Fe 116 mg; Cu 12.5 mg; Zn 60 mg; Co 0.45 g; Ca 1.3 mg; Se 0.3 mg.

**Table 2:** Fatty acid profile of the dietary fats and experimental diets (% of total FAME) (modified from Gasco et al. [9]).

Items	Dietary Fats			Experimental Diets				
	S	H	T	C	H50	H100	T50	T100
C12:0	0.02	48	0.23	0.05	9.12	20.3	0.75	0.30
C14:0	0.08	10.3	2.22	0.09	2.11	4.47	0.97	1.33
C16:0	10.4	12.7	17.6	12.1	15.7	16.1	17.3	18.4
C18:0	4.43	1.90	2.31	2.84	2.62	2.08	2.65	2.22
BCFA	0.01	0.29	0.08	0.24	0.52	0.41	0.38	0.34
C16:1 n-7	0.09	3.20	1.66	0.12	1.31	1.99	0.70	1.04
C18:1 n-9	23	9.11	37.8	20.1	17.3	12.7	24.6	27.3
C18:2 n-6	51.5	9	33.2	52.1	40.9	31	42.9	38.9
C18:3 n-3	7.03	1.01	1.80	7.43	6.79	6.28	5.29	5.51
SFA	15.8	74.8	23.1	16.5	31.5	45.4	23.4	24
UFA	84.2	25.2	76.9	83.5	68.5	54.6	76.6	76
MUFA	25.4	14.1	41.1	23.6	20.5	16.9	27.9	30.9
PUFA	58.8	11.1	35.8	59.9	48	37.7	48.7	45.1
Σn3	7.05	1.17	1.83	7.47	6.82	6.28	5.31	5.51
Σn6	51.6	9.11	33.3	52.3	41	31.1	43.1	39.1

Note: S = soybean oil; H = *Hermetia illucens* fat; T = *Tenebrio molitor* fat; C = control diet; FAME = fatty acid methyl esters; BCFA= branched-chain fatty acids; SFA = saturated fatty acids; UFA = unsaturated fatty acids; MUFA = monounsaturated fatty acids; PUFA = polyunsaturated fatty acids.

**Table 3:** Slaughter traits of the rabbits fed experimental diets (n = 20 rabbits/group).

Items	Experimental Diets					SEM	*p*-Value
	C	H50	H100	T50	T100		
Live weight at slaughter (SW) (g)	2942	2873	2911	2859	2945	28.09	0.727
Chilled Carcass (CC) (g)	1683	1626	1642	1643	1697	17.29	0.668
Dressing out percentage (% SW)	57.2	56.6	56.4	57.6	57.2	0.23	0.529
Head (% CC)	8.24	8.17	8.10	7.91	8.19	0.09	0.783
Liver (% CC)	5.95	5.60	5.35	5.91	5.66	0.08	0.099
Kidneys +thoracic organs (% CC)	3.59	3.47	3.42	3.51	3.32	0.05	0.502
Reference carcass (RC) (g)	1404	1348	1368	1370	1359	20.67	0.937
Perirenal fat (% RC)	2.09	2.18	2.29	2.31	4.73	0.49	0.405

Note: SEM = standard error of the mean; C = control diet; H = *Hermetia illucens* fat; T = *Tenebrio molitor* fat.

**Table 4:** Effect of the experimental diets on the meat quality traits and chemical composition of *Longissimus thoracis* et. *lumborum* of rabbits (n = 15 rabbits/group).

Items	Experimental Diets					SEM	*p*-Value
	C	H50	H100	T50	T100		
**Meat Quality Traits**							
pHu	5.69	5.68	5.71	5.69	5.70	0.01	0.805
L *	58.7	58.2	59.3	59.6	59.6	0.27	0.442
a *	−0.64	−0.40	−0.79	−0.47	−0.62	0.15	0.935
b *	5.62	5.48	5.55	5.52	5.53	0.10	0.995
C *	5.73	5.62	5.75	5.72	5.79	0.09	0.987
H *	96.4	95	98.5	95.6	97.3	1.16	0.964
Thawing losses (%)	11.6	12.8	12	13.5	12.7	0.24	0.105
Cooking losses (%)	28.3	28.3	28.3	28.6	28.3	0.17	0.968
Shear force (Newtons)	30.36	31.71	28.32	29.74	30.64	0.76	0.723
Chemical composition (%)	-	-	-	-	-	-	-
Water	74.8	75	75.1	74.9	75	0.07	0.539
Protein	22.6	22.5	22.6	22.5	22.4	0.05	0.867
Fat	1.20	0.98	0.88	1.18	1.19	0.07	0.493
Ash	1.25	1.29	1.27	1.27	1.25	0.01	0.237

Note: SEM = standard error of the mean; C = control diet; H = *Hermetia illucens* fat; T = *Tenebrio molitor* fat; pHu= ultimate pH; L * = lightness; a * = redness; b * = yellowness; C * = Chroma; H * = hue angle.

**Table 5:** Effect of the experimental diets on the fatty acid profile (% of total FAME), dietary indexes and oxidative status (TBARS, mg MDA/kg of meat) of *Longissimus thoracis* et *lumborum* muscle of rabbits (n = 15 rabbits/group).

Items	Experimental Diets					SEM	*p*-Value
	C	H50	H100	T50	T100		
C12:0	0.20 ^c^	1.22 ^b^	2.53 ^a^	0.20 ^c^	0.19 ^c^	0.11	<0.001
C14:0	2.41 ^d^	4.06 ^b^	5.97 ^a^	2.71 ^c^	2.82 ^c^	0.16	<0.001
C16:0	27.5 ^c^	29.2 ^b^	32.1 ^a^	28 ^b,c^	28.9 ^b,c^	0.29	<0.001
C18:0	6.53	6.36	6.11	6.55	6.28	0.08	0.346
BCFA	0.83 ^b^	0.95 ^a^	0.97 ^a^	0.88 ^b^	0.82 ^b^	0.02	0.002
C16:1 n-7	2.96 ^b^	3.51 ^a,b^	4.01 ^a^	2.86 ^b^	3.45 ^a,b^	0.14	0.048
C18:1 n-9	22.1 ^b^	20.5 ^c^	19.4 ^d^	23.1 ^b^	24.8 ^a^	0.27	<0.001
C18:2 n-6	27.7 ^a^	24.2 ^b,c^	19.4 ^d^	26.3 ^a,b^	23.8 ^c^	0.49	<0.001
C18:3 n-3	3.85 ^a^	3.76 ^a^	3.31 ^b^	3.67 ^a,b^	3.35 ^b^	0.06	0.006
C20:4 n-6	0.57	0.66	0.51	0.55	0.57	0.03	0.545
C20:5 n-3	0.02	0.03	0.03	0.03	0.03	0	0.508
C22:6 n-3	0.04	0.04	0.04	0.04	0.05	0	0.913
SFA ^1^	39.7 ^c^	44.1 ^b^	50 ^a^	40.5 ^c^	41.1 ^c^	0.50	<0.001
UFA ^1^	60.3 ^a^	55.9 ^b^	50 ^c^	59.5 ^a^	58.9 ^a^	0.50	<0.001
MUFA ^1^	27.6 ^a,b^	26.7 ^a,b^	26.2 ^b^	28.4 ^b^	30.7 ^a^	0.34	<0.001
PUFA ^1^	32.7 ^a^	29.2 ^b,c^	23.8 ^d^	31.1 ^a,b^	28.2 ^c^	0.55	<0.001
Σ n-3	3.98 ^a^	3.91 ^a^	3.45 ^b^	3.82 ^a,b^	3.49 ^b^	0.06	0.008
Σ n-6	28.7 ^a^	25.3 ^b,c^	20.3 ^d^	27.3 ^a,b^	24.7 ^c^	0.50	<0.001
Σ n-6/Σ n-3	7.21 ^a^	6.47 ^b^	5.93 ^b^	7.20 ^a^	7.11a	0.09	<0.001
ΣPUFA/ΣSFA	0.85 ^a^	0.68 ^c^	0.49 ^d^	0.79 ^a,b^	0.72 ^b,c^	0.02	<0.001
Total FA (mg/100 g meat)	3146	3387	4282	3178	3166	233	0.492
Dietary indexes and oxidative status							
AI ^2^	0.62 ^d^	0.84 ^b^	1.17 ^a^	0.66 ^c,d^	0.69 ^c^	0.03	<0.001
TI ^2^	0.92 ^c^	1.05 ^b^	1.31 ^a^	0.95 ^c^	1 ^b,c^	0.02	<0.001
PI ^2^	39.6 ^a^	36.3 ^a,b^	29.9 ^c^	37.8 ^a,b^	34.7 ^b^	0.62	<0.001
TBARS (mg MDA/kg meat)	0.39 ^a^	0.24 ^b^	0.27 ^b^	0.23 ^b^	0.22 ^b^	0.02	0.004

Note: SEM = standard error of the mean; C = control diet; H = *Hermetia illucens* fat; T = *Tenebrio molitor* fat; FAME = fatty acid methyl esters; BCFA = branched-chain fatty acids; SFA = saturated fatty acids; UFA = unsaturated fatty acids; MUFA = monounsaturated fatty acids; PUFA = polyunsaturated fatty acids; FA = fatty acids; AI = atherogenicity index; TI = thrombogenicity index; PI = peroxidability index; ^a–d^ Different superscripts within a row indicate significant differences (*p* < 0.05); ^1^ Including minor FAs. ^2^ Calculated as reported by Dal Bosco et al. [22].

**Table 6:** Overall acceptability mean values and rank-sum scores.

Items	Experimental Diets					*p*-Value
	C	H50	H100	T50	T100	
Mean score ^1^	6.8	6.7	6.5	6.7	6.8	0.621
Rank sum ^2^	347.6	377.5	371	357.5	346.5	0.557

Note: C = control diet; H = *Hermetia illucens* fat; T = *Tenebrio molitor* fat; ^1^ Mean scores. Values range from 1, “dislike extremely” to 9, “like extremely”; ^2^ Rank sum scores. Lower rank-sum scores indicate higher overall acceptability, and higher rank-sum scores indicate lower acceptability.
